# A genome-wide association study yields five novel thyroid cancer risk loci

**DOI:** 10.1038/ncomms14517

**Published:** 2017-02-14

**Authors:** Julius Gudmundsson, Gudmar Thorleifsson, Jon K. Sigurdsson, Lilja Stefansdottir, Jon G. Jonasson, Sigurjon A. Gudjonsson, Daniel F. Gudbjartsson, Gisli Masson, Hrefna Johannsdottir, Gisli H. Halldorsson, Simon N. Stacey, Hannes Helgason, Patrick Sulem, Leigha Senter, Huiling He, Sandya Liyanarachchi, Matthew D. Ringel, Esperanza Aguillo, Angeles Panadero, Enrique Prats, Almudena Garcia-Castaño, Ana De Juan, Fernando Rivera, Li Xu, Lambertus A. Kiemeney, Gudmundur I. Eyjolfsson, Olof Sigurdardottir, Isleifur Olafsson, Hoskuldur Kristvinsson, Romana T. Netea-Maier, Thorvaldur Jonsson, Jose I. Mayordomo, Theo S. Plantinga, Hannes Hjartarson, Jon Hrafnkelsson, Erich M. Sturgis, Unnur Thorsteinsdottir, Thorunn Rafnar, Albert de la Chapelle, Kari Stefansson

**Affiliations:** 1 deCODE genetics/AMGEN, 101 Reykjavik, Iceland; 2 Landspitali-University Hospital, 101 Reykjavik, Iceland; 3 Faculty of Medicine, University of Iceland, 101 Reykjavik, Iceland; 4 The Icelandic Cancer Registry, 105 Reykjavik, Iceland; 5 School of Engineering and Natural Sciences, University of Iceland, 101 Reykjavik, Iceland; 6 Division of Human Genetics, Ohio State University Comprehensive Cancer Center, Columbus, Ohio 43210, USA; 7 Department of Cancer Biology and Genetics, Ohio State University Comprehensive Cancer Center, Columbus, Ohio 43210, USA; 8 Division of Endocrinology, Diabetes, and Metabolism, The Ohio State University, Columbus, Ohio 43210, USA; 9 Division of Endocrinology, University Hospital, 50009 Zaragoza, Spain; 10 Division of Medical Oncology, Ciudad de Coria Hospital, 10800 Coria, Spain; 11 Division of Nuclear Medicine, University Hospital, 50009 Zaragoza, Spain; 12 Division of Medical Oncology, Marques de Valdecilla University Hospital, 39008 Santander, Spain; 13 Department of Head & Neck Surgery, The University of Texas MD Anderson Cancer Center, Houston, Texas 77030, USA; 14 Radboud University Medical Centre, Radboud Institute for Health Sciences, 6500HB Nijmegen, The Netherlands; 15 The Laboratory in Mjodd, 109 Reykjavik, Iceland; 16 Department of Clinical Biochemistry, Akureyri Hospital, 600 Akureyri, Iceland; 17 Division of Endocrinology, Department of Internal Medicine, Radboud University Medical Centre, Radboud Institute for Health Sciences, 6500HB Nijmegen, The Netherlands; 18 University of Colorado Hospital, Aurora, Colorado 80045, USA; 19 Department of Pathology, Radboud University Medical Center, Radboud Institute for Molecular Life Sciences, 6500HB Nijmegen, The Netherlands

## Abstract

The great majority of thyroid cancers are of the non-medullary type. Here we report findings from a genome-wide association study of non-medullary thyroid cancer, including in total 3,001 patients and 287,550 controls from five study groups of European descent. Our results yield five novel loci (all with *P*_combined_<3 × 10^−8^): 1q42.2 (rs12129938 in *PCNXL2*), 3q26.2 (rs6793295 a missense mutation in *LRCC34* near *TERC*), 5q22.1 (rs73227498 between *NREP* and *EPB41L4A*), 10q24.33 (rs7902587 near *OBFC1*), and two independently associated variants at 15q22.33 (rs2289261 and rs56062135; both in *SMAD3*). We also confirm recently published association results from a Chinese study of a variant on 5p15.33 (rs2736100 near the *TERT* gene) and present a stronger association result for a moderately correlated variant (rs10069690; OR=1.20, P=3.2 × 10^−7^) based on our study of individuals of European ancestry. In combination, these results raise several opportunities for future studies of the pathogenesis of thyroid cancer.

## Abstract (web summary)

Non-medullary thyroid cancers include papillary and follicular subtypes, and are the most common type of thyroid cancer. Here, the authors extend previous work by performing a large genome-wide association study and find five novel loci associated with this disease.

Thyroid cancer is the most common malignancy of the endocrine system and its incidence in industrialized countries has been rising over the past few decades. At present the incidence rate of thyroid cancer is 7.4 and 22.0 per 100,000 in the United States (US) for males and females of European ancestry, respectively^1^. In Iceland, the incidence rate is 4.1 and 10.7 for males and females, respectively^2^.

Thyroid tumours are classified into four main histology groups: papillary (PTC), follicular (FTC), medullary (MTC) and undifferentiated or anaplastic thyroid carcinomas. The majority of all thyroid tumours are non-medullary; either PTC (80-85%) or FTC (10-15%)^3^,^4^.

Thyroid cancer has been shown to have one of the strongest genetic component of all cancers, and the effect has been shown to extend beyond the nuclear family^5^,^6^,^7^. In an attempt to discover sequence variants conferring risk of thyroid cancer we previously performed two thyroid cancer genome-wide association studies (GWASs) in which we discovered, and published^8^,^9^, five risk variants, located on 2q35, 9q22.33, 8p12 and 14q13.3. These variants have since been widely replicated by other study groups^10^,^11^,^12^,^13^,^14^.

The purpose of our current study was to continue our search for thyroid cancer risk variants by building on our two previous thyroid cancer GWASs. Hence, we have expanded our study group as well as the number of markers analysed. This expansion provided us with both additional marker density and statistical power, resulting in the discovery of five new thyroid cancer risk loci, located at: 1q42.2, 3q26.2, 5q22.1 10q24.33 and 15q22.33. These results highlight several new potential focus points for future thyroid cancer research studies.

## Results

### Imputation and GWAS data

To search for additional thyroid cancer risk loci, we reanalysed our Icelandic GWAS data set after having expanded it close to seven-fold in terms of total number of participants included in the study and approximately doubling the number of variants analysed from our previously reported study^9^. The increase in number of variants is based on whole genome sequencing (WGS) of 15,220 Icelanders to an average depth of 34.5 × (see Methods). The Icelandic non-medullary thyroid cancer GWAS results are based on 1,003 patients and 278,991 controls (see Supplementary Table 1). Apart from previously reported^8^,^9^ variants or their correlates, located on: 2q35, 8p12, 9q22.33 and 14q13.3, no variants met our genome-wide significance criteria (Supplementary Table 2). The threshold for genome-wide significance in the present study was corrected for multiple testing using a weighted Bonferroni procedure based on functional impact of classes of variants^15^ (GWAS significance thresholds range between 2.6 × 10^−7^ and 7.9 × 10^−10^ depending on functional annotations; see Methods).

We then conducted a meta-analysis including the Icelandic results and four additional case-control groups of European descent, with populations from Columbus, Ohio and Houston, Texas, in the United States (US), the Netherlands and Spain. The number of study subjects in the Columbus study group has been increased by over four-fold from the previously reported^9^, and the Houston study group has not been included in any of the previously published GWASs of thyroid cancer. All these study subjects were whole-genome analysed for the first time in the present study by genotyping them using the Illumina Quad array chip genotyping platform. For the Spanish and the two US-study groups we performed genome-wide imputation using the 1000 Genomes Phase 3 Project data. For the Dutch study group, the genome-wide imputation was done using The Genome of the Netherlands^16^ (GoNL) whole-genome sequencing data set (see Methods). In total, the non-Icelandic data set consists of 1,998 patients and 8,559 controls. Per-allele odds ratios and *P* values for all variants in each of the five study group GWAS analyses were obtained using a logistic regression model. There was little evidence of systematic over dispersion of the test statistic (*λ*_GC_=1.021–1.079; Supplementary Fig. 1). A fixed-effects meta-analysis was conducted for 7.1 million variants (with imputation info score≥0.90 in all study groups) in a total of 3,001 thyroid cancer patients and 287,550 controls.

### Association with thyroid cancer

**Figure 1:**
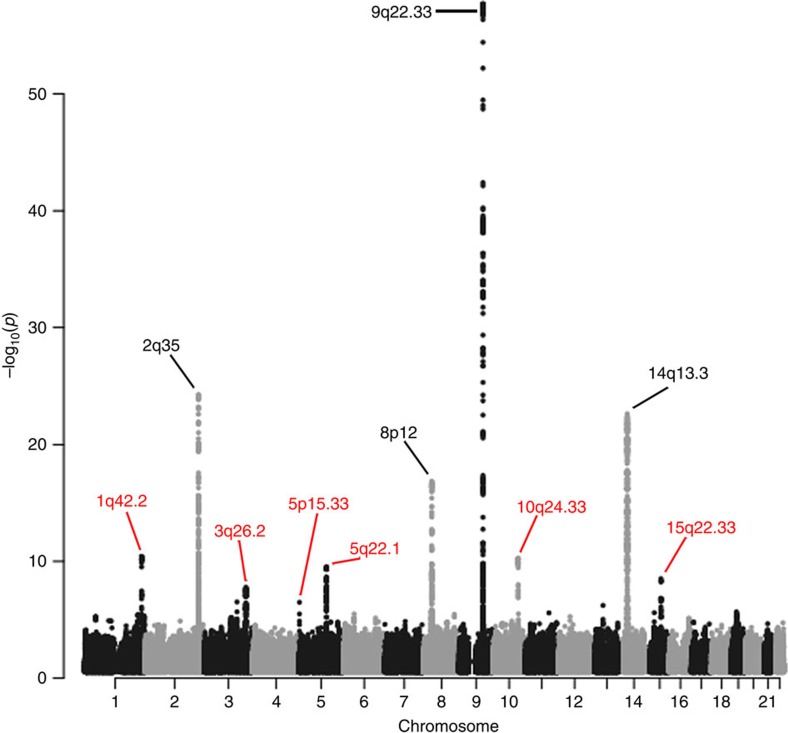
A Manhattan plot of the combined thyroid cancer GWAS results. The Manhattan plot shows 7.1 million variants with high imputation information score (info≥0.90) from the thyroid cancer meta-analysis of GWAS data from 3,001 patients and 287,550 controls from five study groups of European descent coming from Iceland, the Netherlands, Spain and the USA. Shown are negative log_10_-transformed *P* values over 22 autosomes. Previously published risk loci are in black and risk loci reported in current study are in red.

The strongest associations were with previously reported^8^,^9^ thyroid cancer risk variants or with variants in strong linkage disequilibrium (LD) with them, located on: 2q35, 8p12, 9q22.33 and 14q13.3 (Fig. 1, Table 1 and Supplementary Table 2). Only at the 2q35 locus was the variant reported in the present study (rs11693806) significantly stronger than the previously reported rs966423 (results for rs11693806[C] conditioned on rs966423: OR=1.31; *P*=9.5 × 10^−8^; whereas results for rs966423[C] conditioned on rs11693806 are: OR=1.03; *P*=0.61, see Supplementary Table 3). We also discovered five new regions, located on: 1q42.2, 3q26.2, 5q22.1, 10q24.33, 15q22.33, containing variants that are genome-wide significantly associated with thyroid cancer (Table 2 and Supplementary Fig. 2). The 15q22.33 locus contains two variants that associate with thyroid cancer independent of each other (see Table 3).

Unlike the thyroid cancer risk variants previously reported^8^,^9^, none of the variants at these new risk loci is significantly associated (after correcting for multiple testing: *P*<0.01) with serum levels of thyroid-related hormones (that is, thyroid stimulating hormone (TSH), free-triiodothyronine (fT3) or free-thyroxine (fT4); see Supplementary Fig. 3 and Supplementary Table 4). Also, none of the newly discovered variants had a significant association with gender or age at diagnosis. However, the number of samples of the follicular subtype is too few to allow a meaningful comparison of the effect size for different histological subtypes of thyroid cancer.

### Results for other previously reported risk variants

During the preparation of this manuscript, Ge *et al*.^17^ reported results from an association study of thyroid cancer in Chinese, analysing haplotype tagging variants located in the *TERT*-*CLPTM1L* region on 5p15.33; a region reported to contain several variants associated with risk of cancer in several organs. They found a significant association between rs2736100[C] and thyroid cancer in the Chinese. Results in the present study confirm this finding in populations of European ancestry (rs2736100[C]: OR=1.11; *P*=7.3 × 10^−4^, see Supplementary Table 3). However, in our study the strongest association results in this region were for rs10069690[T] (OR=1.20; *P*=3.2 × 10^−7^, see Table 2). Based on the 1000 Genome Project data set, the correlation between rs10069690 and rs2736100 is moderate in both individuals of European ancestry (*r*^2^=0.27) and Asians (*r*^2^=0.26). When we condition the two variants on each other, only results for rs10069690[T] remain significant (*P*=1.3 × 10^−4^ conditioned on rs2736100; compared to *P*=0.11 for rs2736100[C] conditioned on rs10069690, see Supplementary Table 3). Hence, it is likely that the association signals of these two variants reflect the same signal although rs10069690 seems to confer a slight refinement of it when moving from the Chinese to the European ancestry study groups.

For other previously reported^18^,^19^,^20^,^21^ GWAS-identified thyroid cancer risk variants, we nominally replicate results^19^ for rs13184587 located on 5q14.1 (OR=1.08, *P*=0.040; see Supplementary Data set 1).

### Bioinformatics- and eQTL analyses of the novel risk variants

We next performed a bioinformatics- and eQTL (expression quantitative trait locus) analyses, searching for influences of these newly discovered variants on biologically functional elements or gene expression in normal thyroid tissue. Several variants yielded interesting results and had robust association with gene expression (see Supplementary Data set 2). The most interesting findings based on these analyses are discussed below.

At 1q42.2 the lead SNP rs12129938[A] (OR=1.32; *P*=4.0 × 10^−11^) lies within an enhancer in the first intron of *PCNXL2*. Little is known about the function of *PCNXL2* but it is highly conserved and mainly expressed in the brain according to the GTEx Portal (accessed 12 June 2016: http://www.gtexportal.org/home/).

The second most significant variant, rs7902587[T] (OR=1.41; *P*=5.4 × 10^−11^), is located at 10q24.33, approximately16 Kb upstream of *OBFC1*. According to the HaploReg browser (accessed 1 June 2016: http://www.broadinstitute.org/mammals/haploreg/haploreg.php), rs7902587 is located in an enhancer region in fetal brain tissue. Several other variants, highly correlated (*r*^2^≥0.75) with rs7902587, have several hits within both promoters and enhancers in many different tissue types, including the thyroid. OBFC1 is a subunit of an alpha accessory factor (AAF) that stimulates the activity of DNA polymerase-alpha-primase, the enzyme that initiates DNA replication^22^. OBFC1 also appears to function in a telomere-associated protein complex (with C17ORF68 and TEN1 (ref. 23)). rs7902587 also correlates (*r*^2^=0.74) with variants reported^24^,^25^ to associate with longer telomeres as well as with a strongly correlated variant (*r*^2^=0.94) conferring risk of melanoma^26^.

rs73227498[A] (OR=1.37; *P*=3.0 × 10^−10^) at 5q22.1 is located in an intergenic region approximately 394 Kb upstream of *NREP* and 12 Kb downstream of *EPB41L4A*. According to GTEx, *EPB41L4A* has the second highest expression in thyroid of all tissues analysed (see Supplementary Fig. 4). However, the association of the thyroid cancer risk allele (rs73227498[A]) with expression of *EPB41L4A* (*P*=3.0 × 10^−6^) becomes non-significant when conditioned on the most significant cis-eQTL signal (rs821749) for *EPB41L4A* (*P*=0.29 for association of rs73227498 with expression of *EPB41L4A* when conditioned on rs821749). rs821749 is not genome-wide significantly associated with thyroid cancer (*P*=0.0015) in our study. Members of the *EPB41L4A* gene superfamily are thought to regulate the interaction between the cytoskeleton and plasma membrane. This locus also contains other variants strongly correlated (*r*^2^≥0.75) with rs73227498 that are located within regulator elements detected in several different tissues, including from the thyroid (see Supplementary Data set 2).

The 15q22.33 locus contains two independent association signals: rs2289261[C] with OR=1.23; *P*=3.1 × 10^−9^, and rs56062135[T] with OR=1.24; *P*=4.9 × 10^−9^ (see Table 3). The two variants are located intronic in *SMAD3* that is an important transcriptional mediator of transforming growth factor-β (TGF-β) signalling. *SMAD3* is most highly expressed in the thyroid of all tissues analysed according to GTEx (see Supplementary Fig. 5), and the thyroid cancer risk alleles of the two variants associate with increased expression of *SMAD3* in normal thyroid tissue, (rs56062135[T] has *β*=0.46 s.d., *P*=4.8 × 10^−14^, and rs2289261[C] has *β*=0.29 s.d., *P*=1.6 × 10^−6^). According to GTEx, the association between rs56062135 and *SMAD3* expression in thyroid tissue is the strongest cis-eQTL association signal reported for this gene (+/− 1 Mb around transcript start site). A conditional analysis of the expression data, adjusting the two variants for each other, demonstrates that both remain significant, although results for rs56062135 retain more of the significance (rs56062135[T], adjusted for rs2289261, had *β*=0.40 s.d., *P*=4.2 × 10^−10^, and rs2289261[C], adjusted for rs56062135, had *β*=0.14 s.d., *P*=0.021).

At 3q26.2, the lead variant, rs6793295[T] (OR=1.23; *P*=2.7 × 10^−8^), is a missense mutation (p.Ser249Gly) in *LRRC34* and has (either itself or through a highly correlated variant) been previously reported to associate with several diseases and other traits, specifically: pulmonary fibrosis^27^, bladder cancer^28^, multiple myeloma^29^,^30^ and telomere length^25^,^31^,^32^. Little is known about the function of LRRC34 but its role has been suggested to be in ribosome biogenesis in pluripotent stem cells^33^. No significant association with expression of any gene is reported for rs6793295[T] in thyroid tissue, according to GTEx, but the strongest eQTL associations are with decreased expression of *LRRC34* in transformed fibroblasts, tibial tissue and subcutaneous adipose tissue (*β*=−0.35 s.d., −0.39 s.d., and −0.28 s.d., respectively; *P*=1.5 × 10^−8^, 6.7 × 10^−8^ and 7.9 × 10^−8^, respectively). In addition to the lead variant, numerous variants, highly correlated (*r*^2^≥0.75) with rs6793295, are associated with thyroid cancer at this locus (see Supplementary Data set 2). Among them is another missense variant, rs10936600 (p. Leu286Ile), in *LRRC34* and a splice-region variant rs9822885 in *ACTRT3*, a neighbouring gene. Another interesting gene located at this locus is *TERC*, which is the RNA component of the telomerase, involved in maintenance of telomeres. Variants, located at this region and strongly correlated (*r*^2^≥0.95) with the thyroid cancer risk variant, have been reported^31^,^32^ to associate with telomere length and, the allele conferring risk of thyroid cancer is correlated with the allele associated with longer telomeres.

As discussed above, rs10069690[T] (OR=1.20; *P*=3.2 × 10^−7^) is the strongest variant at 5p15.33 in our analysis. It is located in the fourth intron of the *TERT* gene and has previously been reported to confer risk of estrogen receptor negative breast- and ovarian cancer^34^. The authors of that study concluded that the cancer risk was not due to a telomere length association signal also reported by them for moderately correlated variants within this region (rs2736108 and rs7705526 have an *r*^2^ with rs10069690 of 0.04 and 0.34, respectively). Interestingly, the thyroid cancer risk allele of the variant (rs2736100[C]) reported in the Chinese study has also been found to associate with longer telomeres^25^ but that variant is only moderately correlated with the one reported in this region in our study (*r*^2^=0.27 between rs10069690 and rs2736100 in EUR) and therefore it is unclear if telomere length plays a role in conferring risk of thyroid cancer at this locus. Another study^35^ found the thyroid cancer risk allele of rs10069690[T] to be protective of prostate cancer and associated with increased expression of *TERT* in benign prostate tissue. According to GTEx, the expression level of *TERT* in normal thyroid tissue is too low to draw meaningful conclusion. Several other variants at this locus have been associated with numerous diseases, both benign and malignant according to the GWAS Catalog (The NHGRI-EBI Catalog of published GWASs (accessed on 21 June 2016): https://www.ebi.ac.uk/gwas/home).

## Discussion

In summary, our meta-analysis has yielded five new thyroid cancer susceptibility loci. In addition, we confirm a risk locus recently reported^17^ in a study of Chinese thyroid cancer patients. Similarly, as with the bulk of previously reported thyroid cancer risk variants, the majority of these newly discovered variants are located either intronic or intergenic. Recently, the importance of this group of ‘noncoding' variants has been coming clearer, as demonstrated for two of the previously discovered thyroid cancer risk variants, located intergenic at 9q22 and 14q13, and shown to affect the expression and events downstream of long non-coding RNA genes^36^,^37^,^38^. Interestingly, three of the variants reported here are located in regions reported to associate with telomere length and for two of them (rs6793295 on 3q26.2 and rs7902587 on 10q24.33) the allele associated with longer telomeres is correlated with the allele conferring risk of thyroid cancer. For the third variant (rs10069690 on 5p15.33) the correlation is weaker (*r*^2^=0.27 between rs10069690 and rs2736100) even though the direction of the association with thyroid cancer and telomere length is the same as for the other two variants. However, it remains to be determined what biological features account for involvement of these variants in the pathogenesis of non-medullary thyroid cancer.

## Methods

### Study populations

All Icelandic participants in this study are of European ancestry. Icelanders diagnosed with thyroid cancer were identified based on a nationwide list from the Icelandic Cancer Registry (ICR) (http://www.krabbameinsskra.is/indexen.jsp?icd=C73) that contains all Icelandic thyroid cancer patients diagnosed from 1 January 1955, to 31 December 2015. Thereof, 1,108 were non-medullary thyroid cancers. Out of the total list, genotypic information was available from 1,003 (71% are females) individual for the current study and their mean age at diagnosis is 53 years. The 278,991 Icelandic controls (49% females) used in this study consist of individuals belonging to different genetic research projects at deCODE. The controls had a mean age of 57 years. The controls were absent from the nationwide list of thyroid cancer patients according to the ICR. The study was approved by the Data Protection Commission of Iceland and the National Bioethics Committee of Iceland. Written informed consent was obtained from all subjects. Personal identifiers associated with medical information and blood samples were encrypted with a third-party encryption system as previously described^39^.

The Dutch study population consists of 85 non-medullary thyroid cancer cases (70% are females) and 4,956 cancer-free individuals (55% females). The cases were recruited from the Department of Endocrinology, Radboud University Medical Center (Radboudumc), Nijmegen, The Netherlands from November 2009 to June 2010. All patients were of self-reported European descent. Demographic and clinical characteristics were obtained from the patient's medical records. The average age at diagnosis of the patients was 40 years and average age of the controls was 55 years. The DNA for both the Dutch cases and controls was isolated from whole blood using standard methods. The controls were recruited within a project entitled ‘Nijmegen Biomedical Study' (NBS). For a detailed description of this study, see Galesloot *et al*.^40^ The study was approved by the Ethical Committee and the Institutional Review Board of the Radboudumc, Nijmegen, The Netherlands and all study subjects gave written informed consent.

All subjects recruited in Columbus, USA, and included in the current study were of self-reported European descent and provided written informed consent. The 1,580 patients have a mean age of diagnosis of 43 years (74% are females) and were histologically confirmed papillary or follicular thyroid carcinoma (PTC) patients (including traditional PTC and follicular variant PTC). The 1,628 controls have a mean age of 45 years (74% are females) and are individuals without clinically diagnosed thyroid cancer from the central Ohio area. Genomic DNA was extracted from blood. The study was approved by the Institutional Review Board of the Ohio State University.

The study population recruited in Houston, USA, consisted of 250 papillary thyroid cancer (PTC) patients (69% are females) and 363 cancer-free controls (56% are females). The diagnosis of PTC was assigned by histologic review of the surgical or biopsy specimen, and the controls were recruited from among visitors to the institution. All subjects were of self-reported European descent. The mean age of diagnosis of PTC patients was 45 years and the mean age of controls at recruitment was 53 years. Genomic DNA was extracted from buffy coat cells isolated from peripheral blood. The study was approved by the MD Anderson Cancer Center Institutional Review Board and each subject provided written informed consent.

The Spanish study population consisted of 83 non-medullary thyroid cancer cases. The cases were recruited from the Oncology Department of Zaragoza Hospital in Zaragoza, Spain, from October 2006 to June 2007. All patients were of self-reported European descent. Clinical information including age at diagnosis, grade and stage was obtained from medical records. The average age at diagnosis for the patients was 49 years (71% are females). The 1,612 Spanish control individuals (51% are females) had a mean age of 47 and were approached at the University Hospital in Zaragoza, Spain, and were not known to have thyroid cancer. The DNA for both the Spanish cases and controls was isolated from whole blood using standard methods. Study protocols were approved by the Institutional Review Board of Zaragoza University Hospital. All subjects gave written informed consent.

### Thyroid-related hormone measurements

TSH, fT_4_ and fT_3_ levels were measured in blood samples of Icelanders seeking medical care between the years 1997 and 2015 at the Landspitali University Hospital, the Clinical Laboratory in Mjodd, Reykjavik, Iceland, and Akureyri Hospital, Akureyri, Iceland. The QTL association results are based on measurements for the following number of Icelanders: TSH=188,057 individuals; fT3=51,047 individuals; fT4=120,879 individuals. The measurements were normalized to a standard normal distribution using quantile-quantile normalization and then adjusted for centre, gender, year of birth and age at measurement. For individuals for which more than one measurement was available we used the average of the adjusted normalized values. A generalized form of linear regression that accounts for the relatedness between individuals was used to test for the association of quantitative traits (that is, TSH, fT3 and fT4), with sequence variants.

### Genotyping and statistical methods

The Icelandic thyroid cancer GWAS data set used in the current study is based on WGS, chip genotyping and long-range phasing of Icelandic population samples^41^. In brief, we whole genome sequenced 15,220 Icelanders using Illumina technology (Illumina, San Diego, CA, USA) to an average depth of at least 34 × , resulting in the identification of some 94 million variants. Using imputation assisted by long-range haplotype phasing^42^,^43^ and after removing variants with imputation information content below 0.8 as well as with an imputed minor allele frequency below 0.01%, we successfully inferred the genotypes of 32,463,443 variants in 434,571 Icelanders, of whom 151,677 had been genotyped using the Illumina chip genotyping platform. The remaining 282,894 Icelanders are first- and second-degree relatives of the chip-typed individuals and are imputed by aid of genealogic information. Of the patients used in this study 639 (64%) were chip genotyped and the remaining 364 were imputed by aid of genealogical information. Comparable numbers for the controls used are 126,251 (45%) chip genotyped and the remaining 152,739 were imputed by aid of genealogical information. In the meta-analysis only variants with imputation info score≥0.9 were included.

The Dutch, Spanish and US study samples were genotyped using Omni-1 Quad-bead chips (Illumina, San Diego, CA, USA). For each sample set, variants were excluded if they (i) had<94% yield, (ii) had<1% MAF, (iii) failed Hardy-Weinberg test (*P*<1 × 10^−6^) or (iv) showed significant (*P*<1 × 10^−6^) difference between genotype batches. Samples with<94% yield were excluded. The resulting genotypes were phased using SHAPEIT (v2.790) (ref. 44), and used to impute un-genotyped variants using IMPUTE2 (v2.3.2) (ref. 45). All but the Dutch samples were imputed using the 1000 Genomes Phase 3 reference data (October 2014 release) that includes phased genotypes for about 80 million variants and for 2,504 individuals of various ethnicities^46^. The Dutch study samples were imputed using the GoNL (ref. 16) data set generated by whole-genome sequencing of 249 Dutch trios (498 unrelated parents and 249 children).

### Single track assay SNP genotyping

For validation purposes we directly genotyped the variants reported in Table 2 of the main text for a sub-set of 2,754 cases and controls form all five case-control groups of the present study. The correlation (*r*^2^) between imputed and directly genotyped was above 0.92 for all markers. Due to low imputation information score (ranging between 0.72 and 0.78) for s116909374 on 14q13.3 in the non-Icelandic study groups, this variant was directly genotyped in all available samples and the results combined with the Icelandic imputation results (which had imputation score=1.0) and presented in Table 1 and in Supplementary Table 2, The direct genotyping was carried out by deCODE Genetics in Reykjavik, Iceland, applying the Centaurus^47^ (Nanogen) platform.

### Population structure

To study the population structure and the ancestry of samples in the Dutch, Spanish and US cohorts we used the ADMIXTURE (v 1.2) (ref. 48) and EIGENSOFT (v 6.0.1) (ref. 49) software. Samples were excluded if they were identified as ethnic outliers in the respective cohort, and to adjust for remaining population substructure ten principle components were included as covariates in the subsequent association analysis. Likewise, for the Icelandic cohort population, substructure was adjusted for by including county of origin as a covariate in the analysis.

### Association testing

Logistic regression was used to test for association between variants and disease, assuming a multiplicative model, treating disease status as the response and expected genotype counts from imputation as covariates. For the Icelandic cohort this was done using software developed at deCODE genetics^41^, but the Dutch, Spanish and US cohorts were analysed using the SNPTEST (v.2.5) software^50^. Testing was performed using the likelihood ratio statistic. For the Icelandic study group patients and controls are matched on gender and age at diagnosis or age at inclusion but for the other study groups we did not adjust for these factors. Variants in the 1000 Genomes and GoNL imputation data sets were mapped to NCBI Build38 positions and matched to the variants in the Icelandic data set based on allele variation. Results from the different study groups were combined using a Mantel-Haenszel model^51^ in which the groups were allowed to have different population frequencies for alleles and genotypes but were assumed to have a common OR. Heterogeneity was tested by comparing the null hypothesis of the effect being the same in all populations to the alternative hypothesis of each population having a different effect using a likelihood ratio test. *I*^2^ lies between 0 and 100% and describes the proportion of total variation in study estimates that is due to heterogeneity.

### Association significance thresholds

The threshold for genome-wide significant association in the current study was corrected for multiple testing using a class-specific Bonferroni procedure based on functional impact of classes of variants^15^. This yielded significance thresholds of: (i) 2.6 × 10^−7^ for 8,474 high-impact variants (comprised of: stop-gained, frameshift, splice acceptor or donor), (ii) 5.1 × 10^−8^ for 149,983 moderate-impact variants (comprised of: missense, splice-region variants and in-frame INDELs), (iii) 4.6 × 10^−9^ for 2,283,889 low impact variants (comprised of: synonymous variants 3′ and 5′ UTR variants), (iv) 2.3 × 10^−9^ for 3,913,058 intergenic and deep intronic variants overlapping DNase hypersensitivity sites and (v) 7.9 × 10^−10^ for 26,108,039 other variants (intergenic and deep intronic).

### Genomic control adjustment

To account for inflation in test statistics due to cryptic relatedness and stratification within the case and control sample sets, we applied the method of LD score regression^52^. With a set of 1.1million variants we regressed the *χ*^2^ statistics from our GWAS scan against LD score and used the intercept as a correction factor. The LD scores were downloaded from an LD score database (accessed 23 June2015: ftp://atguftp.mgh.harvard.edu/brendan/1k_eur_r2_hm3snps_se_weights.RDS). The estimated correction factor was 1.079 for the Icelandic samples, mostly due to relatedness of the individuals; all *P* values in the Icelandic GWAS study were adjusted for this inflation. For the other study groups, the genomic adjustment factors were modest, in all cases below 1.043, and this was not adjusted for. For the combined meta-analysis, the correction factor was estimated to be 1.031.

### Bioinformatics analysis

To inspect if the thyroid cancer risk variants might have regulatory effects or be located within regions with predicted biological features we performed an analysis selecting the lead variant at each of the newly discovered risk loci as well as all variants strongly correlated (*r*^2^≥0.75) with the lead variant. For this set of variants, we searched for overlaps with known regulatory regions as follows: first we used ENSEMBL to determine whether the variant had been assigned a regulatory region ENSR number. Then we examined the ENCODE data and looked for any evidence of ChIP-Seq transcription factor binding and DNaseI hypersensitivity sites^53^,^54^. We also looked for enhancer and promoter chromatin segmentation states using the 25-state HMM from the Roadmap consortium^55^. Then we looked for correlations between DNaseI hypersensitive sites and local gene expression using results described by Sheffield *et al*.^56^. Finally, we searched for eQTL associations between variants and genes using publicly available data^57^,^58^, and gene targets of distal enhancers using looping chromatin^59^.

### Expression analysis

Gene expression and genotype data for 278 thyroid tissues from the GTEx Release phs000424.v6. p1 from 5.10.2015 (GTEx Analysis V6) were downloaded from the GTEx consortium^57^ via dbGAP. Linear regression analysis for expression of *SMAD3* and genotypes of rs73227498 and rs821749 on 5q22.1 as well as for rs2289261 and rs56062135 on 15q22.33 was performed using quantile normalized expression values and the following covariates from GTEx: 3 genotyping principal components, gender, genotyping platform (Illumina's OMNI 5M or 2.5M array) and 35 PEER factors.

### Data availability

The Icelandic population WGS data have been deposited at the European Variant Archive under accession code PRJEB8636. The authors declare that the data supporting the findings of this study are available within the article, its Supplementary Information files and on request.

## Additional information

**How to cite this article:** Gudmundsson, J. *et al*. A genome-wide association study yields five novel thyroid cancer risk loci. *Nat. Commun.* **8,** 14517 doi: 10.1038/ncomms14517 (2017).

**Publisher's note:** Springer Nature remains neutral with regard to jurisdictional claims in published maps and institutional affiliations.

## Supplementary Material

Supplementary Information Supplementary Figures, Supplementary Tables, and Supplementary References

Supplementary Data 1 Results from meta-analysis for GWAS-identified risk variants by other groups

Supplementary Data 2 Functional annotation of lead and correlated variants at GWAS risk loci (r2=0.75 in Icelandic population) from Haploreg, ENCODE, Ensembl, GtEX, TargetFinder, Blood eQTL browser and Regulatory Elements Database

## Figures and Tables

**Table 1:** Results for the strongest variants in the current study for the five previously reported thyroid cancer risk loci.

**Locus**	Marker* **(EA/OA)**	**Position (bp)**	**Annotation**	**EAF**	**Allelic OR (95% CI)**	***P*** **value**	**Pairwise correlation with previously reported SNP (*****r***^**2**^**)**
2q35	rs11693806 (C/G)*P*_het_=0.0069; *I*^2^=71.7	217,427,435	Non-coding transcript exon variant	0.319	1.43(1.33, 1.54)	1.5 × 10^−24^	0.47 with rs966423
8p12	rs2466076 (G/T)*P*_het_=0.41; *I*^2^=0	32,575,278	Intron variant	0.484	1.32(1.23, 1.41)	1.5 × 10^−17^	0.94 with rs2439302
9q22.33	rs1588635 (A/C)*P*_het_=0.48; *I*^2^=0	97,775,520	Intergenic variant	0.396	1.69(1.59, 1.82)	2.0 × 10^−58^	0.99 with rs965513
14q13.3	rs368187 (G/C)*P*_het_=0.089; *I*^2^=50.5	36,063,370	Non-coding transcript exon variant	0.581	1.39(1.30, 1.47)	5.1 × 10^−23^	0.70 with rs944289
14q13.3	rs116909374 (T/C)*P*_het_=0.85; *I*^2^=0	36,269,155	Intergenic variant	0.034	1.81(1.57, 2.08)	1.1 × 10^−16^	same as current SNP

Shown is: the chromosomal locus, the risk markers and the effect allele (EA), the other allele (OA), the position of the marker in base pairs (bp) with reference to Build 38, the functional annotation (Annotation) according to Variant Effect Predictor (VEP) (ref. 60), the average unweighted effect allele frequency (EAF) in controls, the allelic odds ratio (OR) for the effect allele with 95% confidence interval (CI), the *P* value and the correlation coefficient (*r*^2^) between the marker reported in the present study and the marker previously reported^8^,^9^ for each of the five loci. Association testing was performed using the likelihood ratio statistic and results from different study groups were combined using a Mantel-Haenszel model (see Methods).

Note that marker rs116909374 on 14q13.3 is still the strongest association signal at that locus in the current meta-analysis. Shown is the *P* value for the heterogeneity (*P*_het_) between the different study groups and the heterogeneity statistic (*I*^2^) representing the fraction of variability due to heterogeneity between study groups.

^*^For all markers but rs116909374, was the imputation information score ≥0.97 and the association results shown are imputed results from the meta-analysis of the Icelandic, US (Columbus, Ohio and Houston, Texas), Dutch and Spanish study groups. For rs116909374 the combined results are made up of imputed data from Iceland (with imputation information score=1.0) and directly generated genotypes for the other four study groups.

**Table 2:** Association results for five new genome-wide significant loci and the variant replicating the 5p15.33 locus.

**Locus**	**Lead marker (EA/OA)**	**Position (bp)**	**Annotation/Nearby gene(s)**	**EAF**	**Study group**	**Allelic OR (95% CI)**	***P*** **value**
1q42.2	rs12129938 (A/G)	233,276,815	Intron variant *PCNXL2*	0.783	Iceland	1.37 (1.20, 1.54)	9.1 × 10^−7^
	*P*_het_=0.73; *I*^2^=0			0.810	Columbus, USA	1.23 (1.10, 1.41)	5.9 × 10^−4^
				0.808	Houston, USA	1.28 (0.96, 1.70)	0.091
				0.786	The Netherlands	1.28 (0.88, 1.85)	0.19
				0.789	Spain	1.56 (1.06, 2.27)	0.024
				0.795	All combined	1.32 (1.20, 1.43)	4.0 × 10^−11^
3q26.2	rs6793295 (T/C)	169,800,667	Missense variant *TERC, LRRC34*	0.780	Iceland	1.30 (1.16, 1.47)	1.9 × 10^−5^
	*P*_het_=0.68; *I*^2^=0			0.755	Columbus, USA	1.19 (1.06, 1.33)	0.0018
				0.764	Houston, USA	1.28 (0.98, 1.67)	0.068
				0.734	The Netherlands	1.27 (0.90, 1.79)	0.18
				0.760	Spain	1.02 (0.70, 1.47)	0.93
				0.759	All combined	1.23 (1.15, 1.33)	2.7 × 10^−8^
5p15.33	rs10069690 (T/C)	1,279,675	Intron variant *TERT*	0.259	Iceland	1.25 (1.12, 1.40)	4.8 × 10^−5^
	*P*_het_=0.49; *I*^2^=0			0.277	Columbus, USA	1.12 (1.00, 1.25)	0.043
				0.306	Houston, USA	1.21 (0.94, 1.57)	0.14
				0.247	The Netherlands	1.38 (0.97, 1.96)	0.074
				0.284	Spain	1.40 (0.99, 1.98)	0.059
				0.275	All combined	1.20 (1.12, 1.29)	3.2 × 10^−7^
5q22.1	rs73227498 (A/T)	112,150,207	Intergenic variant *NREP, EPB41L4A*	0.855	Iceland	1.35 (1.16, 1.56)	8.1 × 10^−5^
	*P*_het_=0.30; *I*^2^=17.7			0.891	Columbus, USA	1.39 (1.20, 1.61)	1.4 × 10^−5^
				0.898	Houston, USA	1.67 (1.19, 2.33)	0.0030
				0.849	The Netherlands	0.92 (0.60, 1.41)	0.69
				0.869	Spain	1.28 (0.81, 2.04)	0.29
				0.872	All combined	1.37 (1.23, 1.49)	3.0 × 10^−10^
10q24.33	rs7902587 (T/C)	103,934,543	Intergenic variant *OBFC1*	0.095	Iceland	1.40 (1.21, 1.63)	1.0 × 10^−5^
	*P*_het_=0.42; *I*^2^=0			0.120	Columbus, USA	1.32 (1.12, 1.55)	8.2 × 10^−4^
				0.138	Houston, USA	1.52 (1.06, 2.19)	0.024
				0.096	The Netherlands	2.23 (1.34, 3.73)	0.0022
				0.094	Spain	1.44 (0.83, 2.48)	0.19
				0.109	All combined	1.41 (1.27, 1.56)	5.4 × 10^−11^
15q22.33	rs2289261 (C/G)	67,165,147	Intron variant *SMAD3*	0.669	Iceland	1.17 (1.05, 1.30)	0.0037
	*P*_het_=0.24; *I*^2^=26.7			0.700	Columbus, USA	1.33 (1.19, 1.47)	1.9 × 10^−7^
				0.703	Houston, USA	1.30 (1.02, 1.66)	0.037
				0.666	The Netherlands	1.14 (0.82, 1.58)	0.43
				0.678	Spain	0.95 (0.68, 1.33)	0.77
				0.683	All combined	1.23 (1.15, 1.32)	3.1 × 10^−9^

Position is according to Build 38 of the reference genome. Shown is the effect allele (EA), the other allele (OA), the effect allele frequency in controls (EAF), the allelic odds ratio (OR) for the effect allele with upper and lower 95% confidence intervals (CI) and the *P* value for association testing between variants and disease which was performed using the likelihood ratio statistic. Results from different study groups were combined using a Mantel-Haenszel model (see Methods). Annotation is according to Variant Effect Predictor (VEP) (ref. 60). Shown are also the *P* value for the heterogeneity (*P*_het_) between the different study groups and the heterogeneity statistic (*I*^2^) representing the fraction of variability due to heterogeneity between study groups. All markers listed in table had imputation information score≥0.95.

**Table 3:** Results from the conditional analysis of the 15q22.33 locus showing two independent association signals.

**Marker (EA/OA)**	**Position (bp)**	**EAF**	**Study group**	**Unadjusted results for rs2289261_C**		**Results for rs2289261_C adjusted on rs56062135**	
				**Allelic OR (95% CI)**	***P*** **value**	**Allelic OR (95% CI)**	***P*** **value**
rs2289261 (C/G)	67,165,147	0.669	Iceland	1.17 (1.05, 1.30)	0.0037	1.08 (0.97, 1.21)	0.17
		0.700	Columbus, USA	1.33 (1.19, 1.47)	1.9 × 10^−7^	1.27 (1.13, 1.43)	5.3 × 10^−5^
		0.703	Houston, USA	1.30 (1.02, 1.66)	0.037	0.87 (0.39, 1.92)	0.73
		0.666	The Netherlands	1.14 (0.82, 1.58)	0.43	1.09 (0.77, 1.55)	0.63
		0.678	Spain	0.95 (0.68, 1.33)	0.77	0.78 (0.53, 1.14)	0.20
		0.683	All combined	1.23 (1.15, 1.32)	3.1 × 10^−9^	1.14 (1.06, 1.23)	6.6 × 10^−4^
				*P*_het_=0.24; *I*^2^=26.7		*P*_het_=0.072; *I*^2^=53.5	
				**Unadjusted results for rs56062135_T**		**Results for rs56062135_T adjusted on rs2289261**	
				**Allelic OR (95% CI)**	***P*** **value**	**Allelic OR (95% CI)**	***P*** **value**
rs56062135 (T/C)	67,163,292	0.231	Iceland	1.23 (1.10, 1.37)	2.2 × 10^−4^	1.19 (1.05, 1.34)	0.0049
		0.279	Columbus, USA	1.24 (1.11, 1.39)	2.2 × 10^−4^	1.11 (0.98, 1.25)	0.093
		0.271	Houston, USA	1.25 (0.95, 1.64)	0.11	0.62 (0.23, 1.66)	0.34
		0.239	The Netherlands	1.17 (0.82, 1.67)	0.39	1.12 (0.77, 1.62)	0.55
		0.243	Spain	1.50 (1.04, 2.16)	0.029	1.66 (1.12, 2.47)	0.012
		0.253	All combined	1.24 (1.16, 1.34)	4.9 × 10^−9^	1.16 (1.07, 1.26)	3.1 × 10^−4^
				*P*_het_=0.89; *I*^2^=0		*P*_het_=0.25; *I*^2^=26.3	

Shown are association results from the meta-analysis before and after conditional analysis, the marker and the corresponding effect allele (EA) and the other allele (OA), the genomic position in base pairs (bp) with reference to Build 38, the allelic odd ratio (OR) of the effect allele with 95% confidence intervals (CI) and the *P* value generated using the likelihood ratio statistic. Results from different study groups were combined using a Mantel-Haenszel model (see Methods) The correlation (*r*^2^) in Iceland between rs2289261 and rs56062135 is 0.15. Shown is the *P* value for the heterogeneity (*P*_het_) between the different study groups and the heterogeneity statistic (*I*^2^) representing the fraction of variability due to heterogeneity between study groups. Both markers in table had imputation information score≥0.99.
