# Supplementary material for: A genome-wide association study yields five novel thyroid cancer risk loci
Source: Nat Commun. 2017 Feb 14;8:14517. doi: 10.1038/ncomms14517 (PMC5316879; doi:10.1038/ncomms14517)
Supplement: Supplementary Information — Supplementary Figures, Supplementary Tables, and Supplementary References [file ncomms14517-s1.pdf]

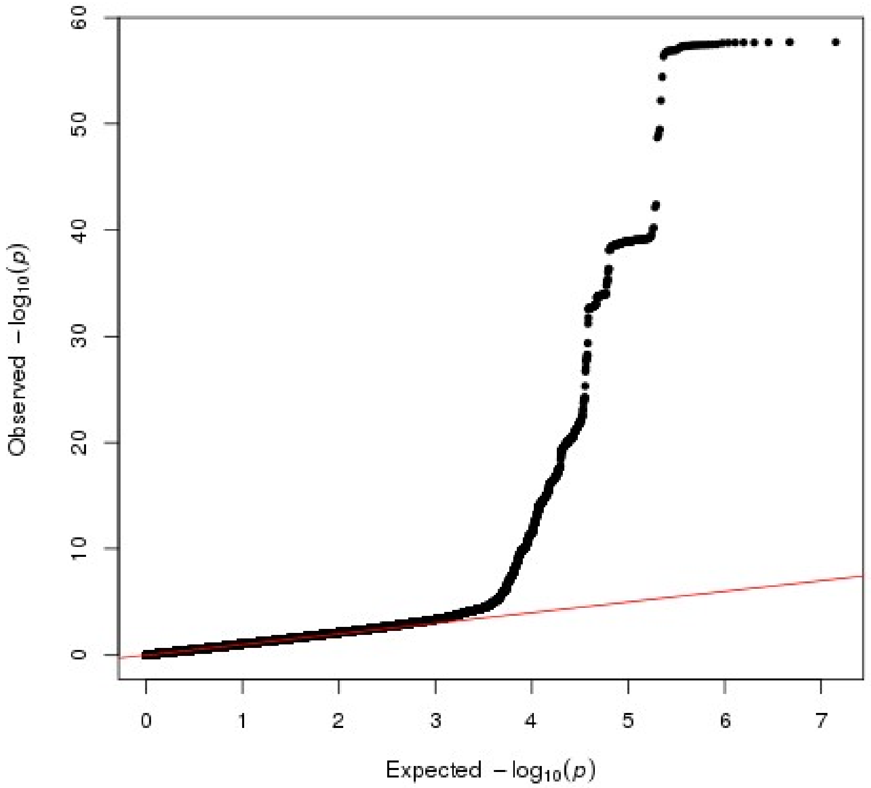

Supplementary Figure 1. Quantile-quantile plot for the 7.1 million chi-square statistics with high-info content from the meta-analysis. The read diagonal line represents expected distribution assuming no inflation of the chi-square statistics.

a

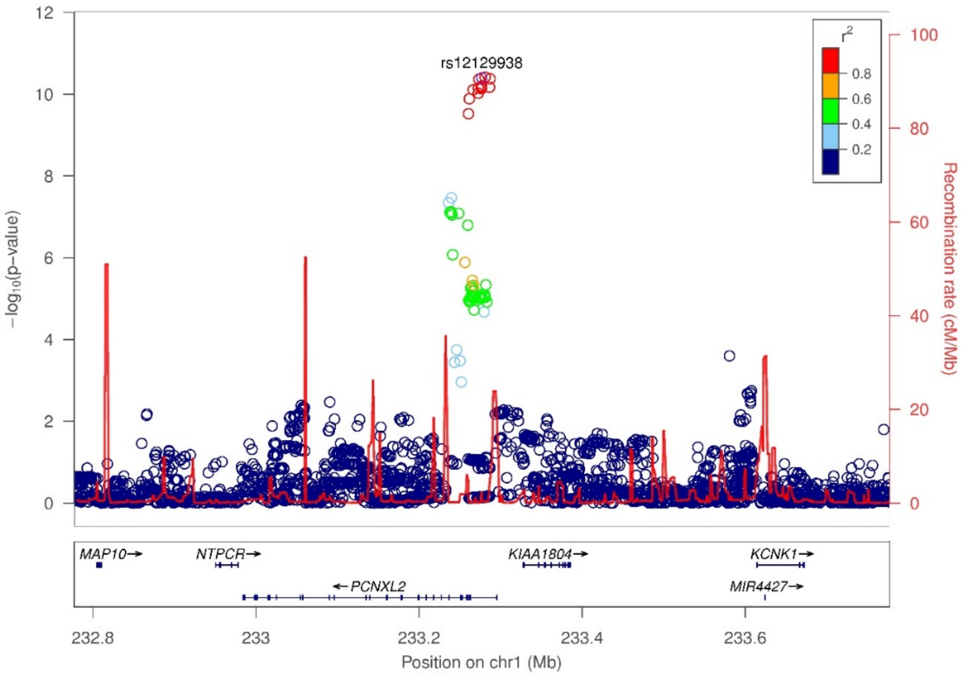

b

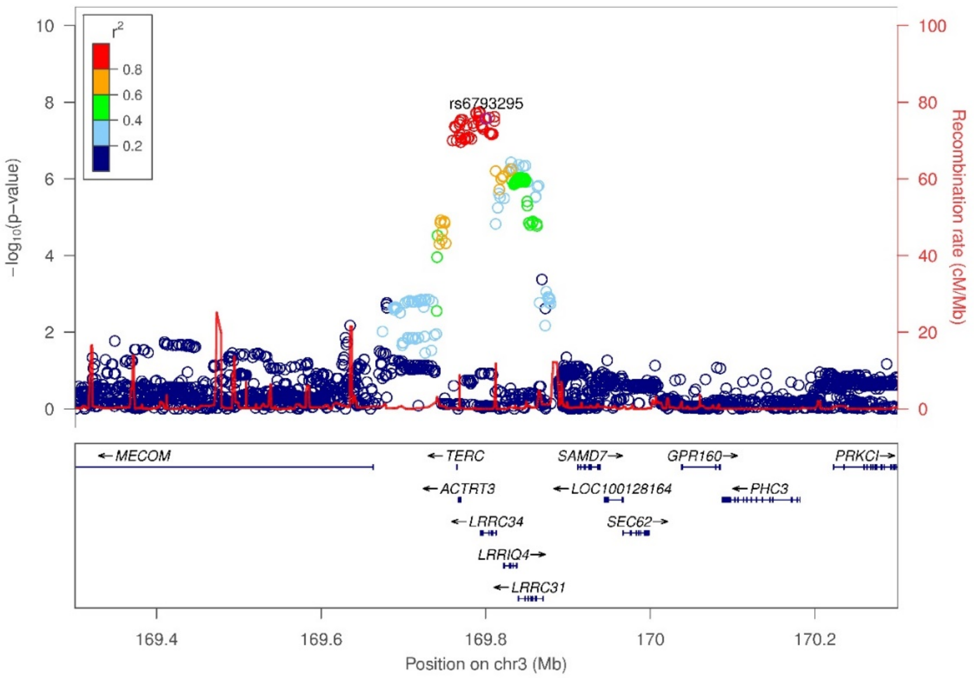

Supplementary Figure 2.

c

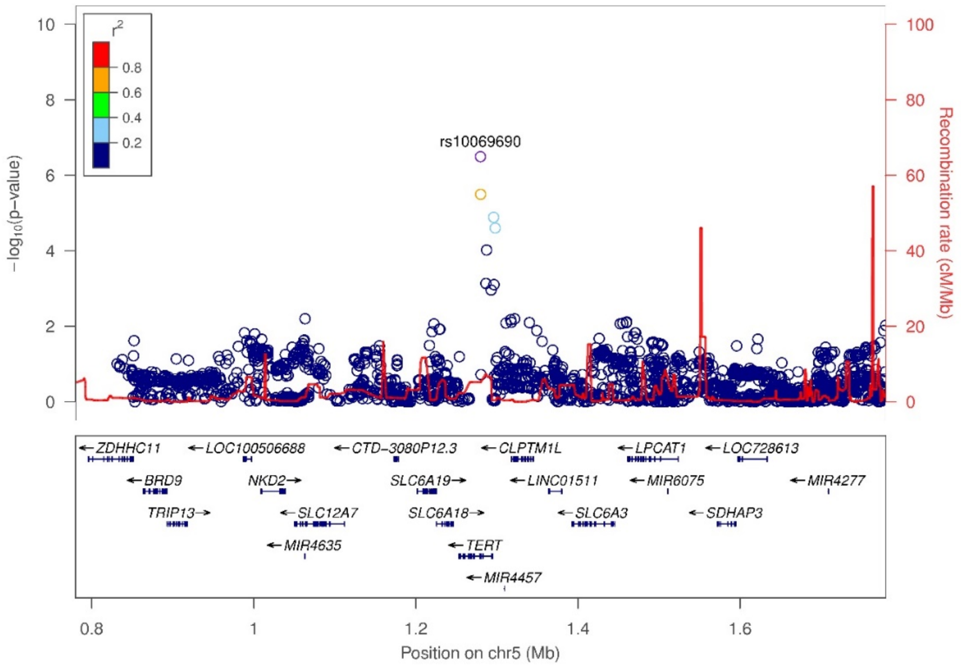

d

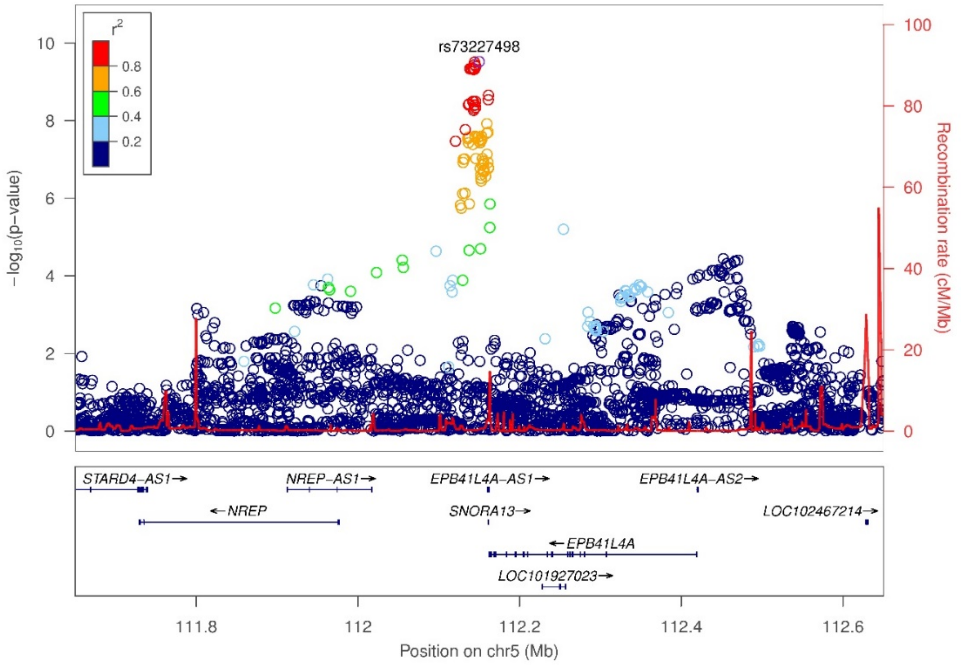

Supplementary Figure 2. Cont.

e

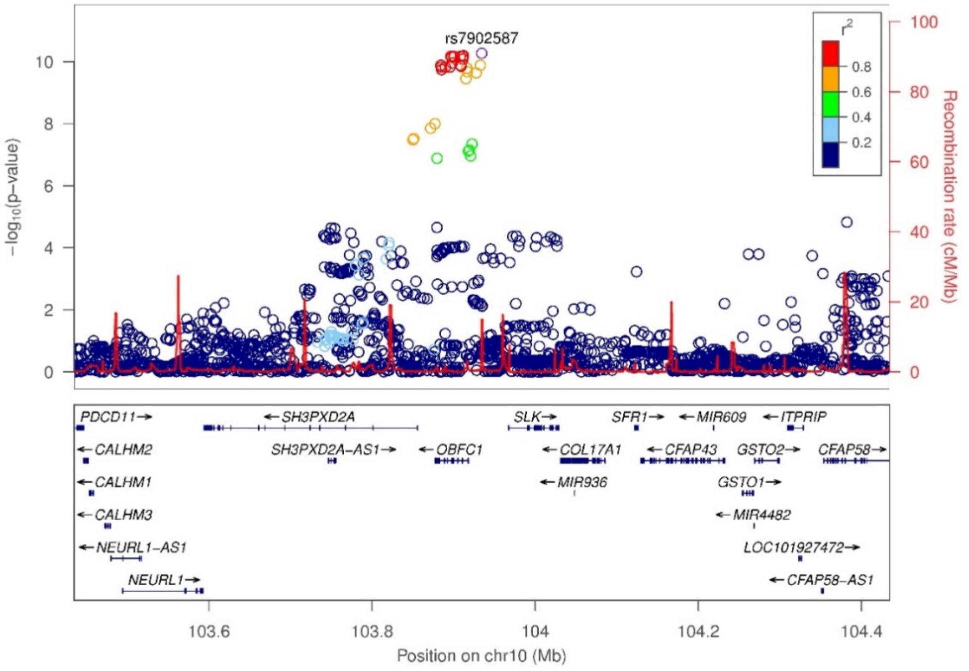

f

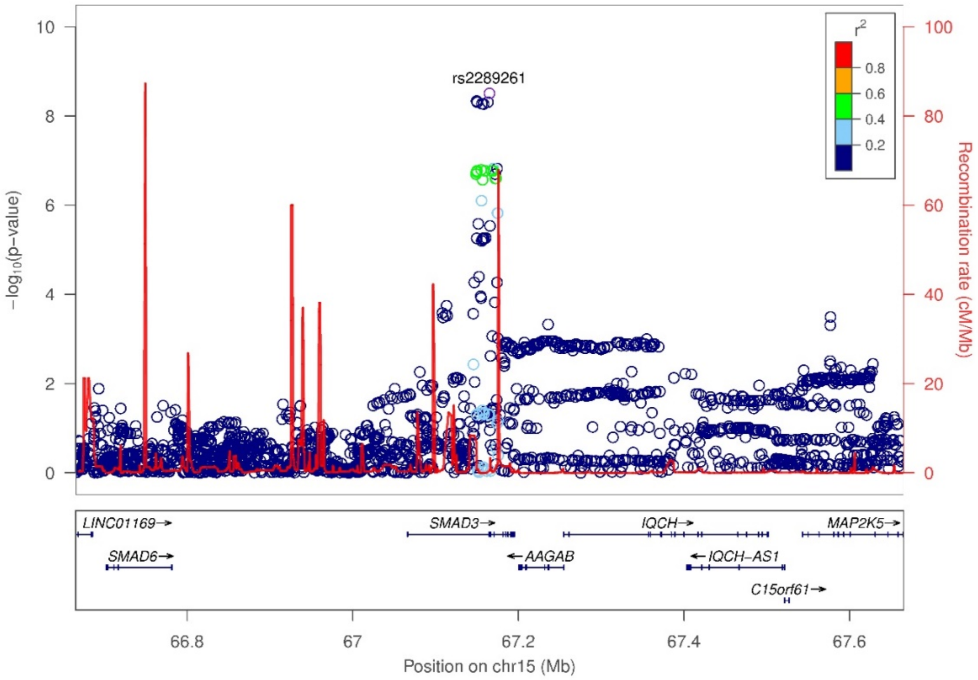

Supplementary Figure 2. Cont.

g

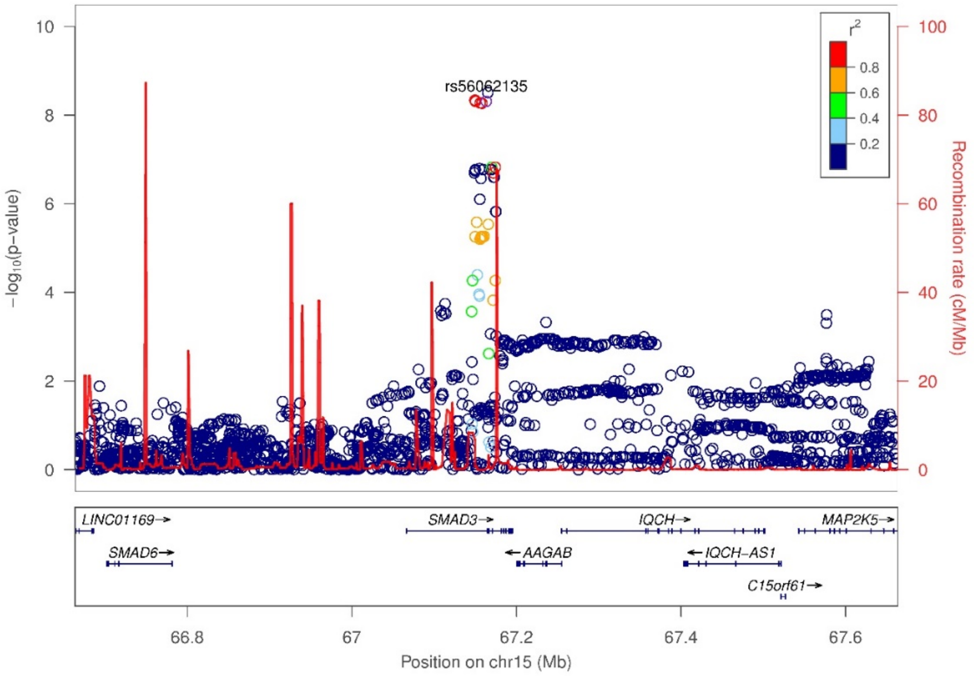

Supplementary Figure 2. Cont. Regional association plot for variants with imputation information score ≥0.90 and located at one of the newly discovered thyroid cancer risk loci. Shown are the negative log_{10}-transformed *P*-values from the meta-analysis The risk loci are located on: 1q42.2 (**a**), 3q26.2 (**b**), 5p15.33 (**c**), 5q22.1 (**d**), 10q24.33 (**e**), and 15q22.33 (**f** and **g**). The lead marker at each locus is represented by a purple circle and other circle-colors indicate linkage disequilibrium (LD) with lead marker (LD is represented as the correlation coefficient (r^2) calculated based on the Icelandic whole-genome sequencing dataset. The red line shows the recombination rate from Phase 2 HapMap estimated from phased haplotypes in HapMap Release 22 (Ref.[1]) estimated from the CEU, YRI and JPT+CHB populations, and mapped onto NCBI hg38, Build38. The plot was created using a standalone version of the LocusZoom software[2]

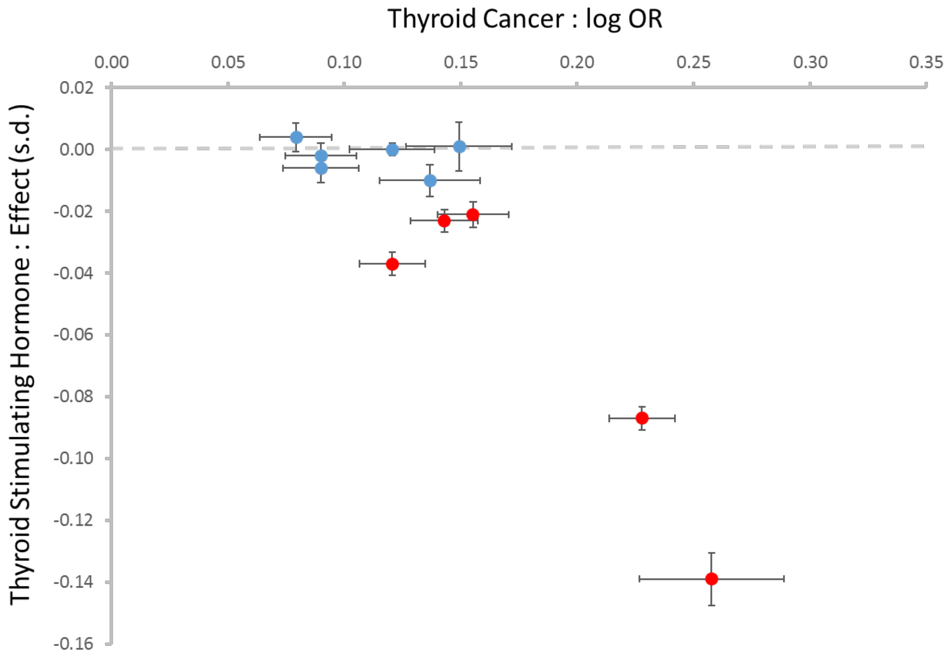

Supplementary Figure 3. Shown is the QTL association effect in standard deviation units (s.d.) for serum levels of thyroid stimulating hormone (the y-axis) and the log of the odds ratio (OR) from the association analysis of thyroid cancer (the x-axis); for previously published[3,4] thyroid cancer risk variants (filled red circles), located on 2q35, 8p12, 9q22.33 and 14q13.3, as well as for variants reported in current study (filled blue circles), located on 1q42.2, 3q26.2, 5q22.1, 10q24.33, 15q22.33. The error bars for each data-point in the figure denote plus or minus (+/-) one standard error. The broken horizontal line indicates the cero on the y-axis. The data used to generate this figure can be found in Table 2 of the main text, Supplementary Table 2 and Supplementary Table 4.

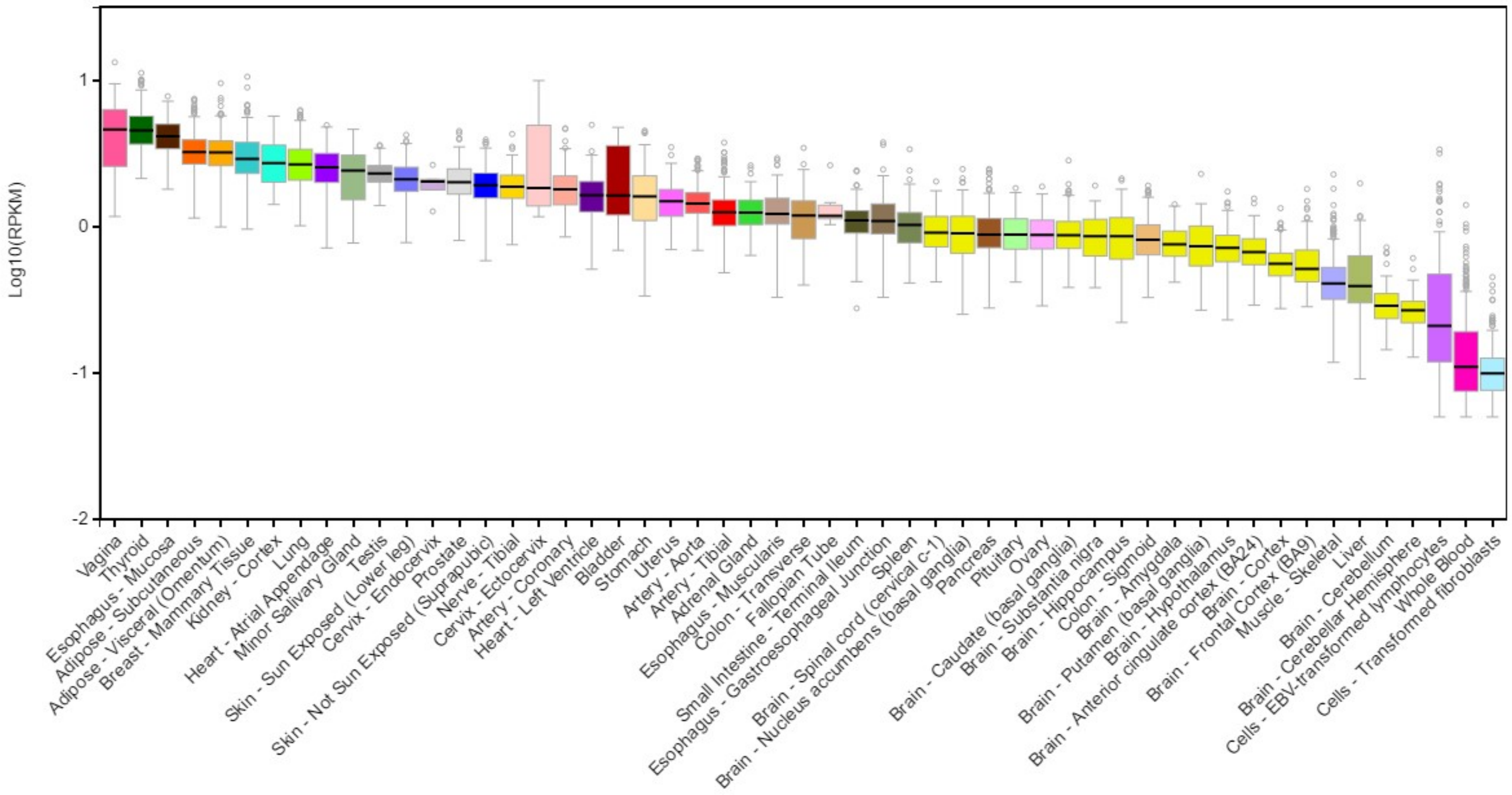

Supplementary Figure 4. Expression levels of *EPB41L4A* in a collection of human tissues from the GTEx consortium

Expression values are shown in RPKM (Reads Per Kilobase of transcript per Million mapped reads), calculated from a gene model with isoforms collapsed to a single gene. No other normalization steps have been applied. Box plots are shown as median and 25th and 75th percentiles; points are displayed as outliers if they are above or below 1.5 times the interquartile range indicated by the whiskers. For further details, see the GTEx homepage: **(**date accessed July 6th, 2016; http://www.gtexportal.org/home/**)**.

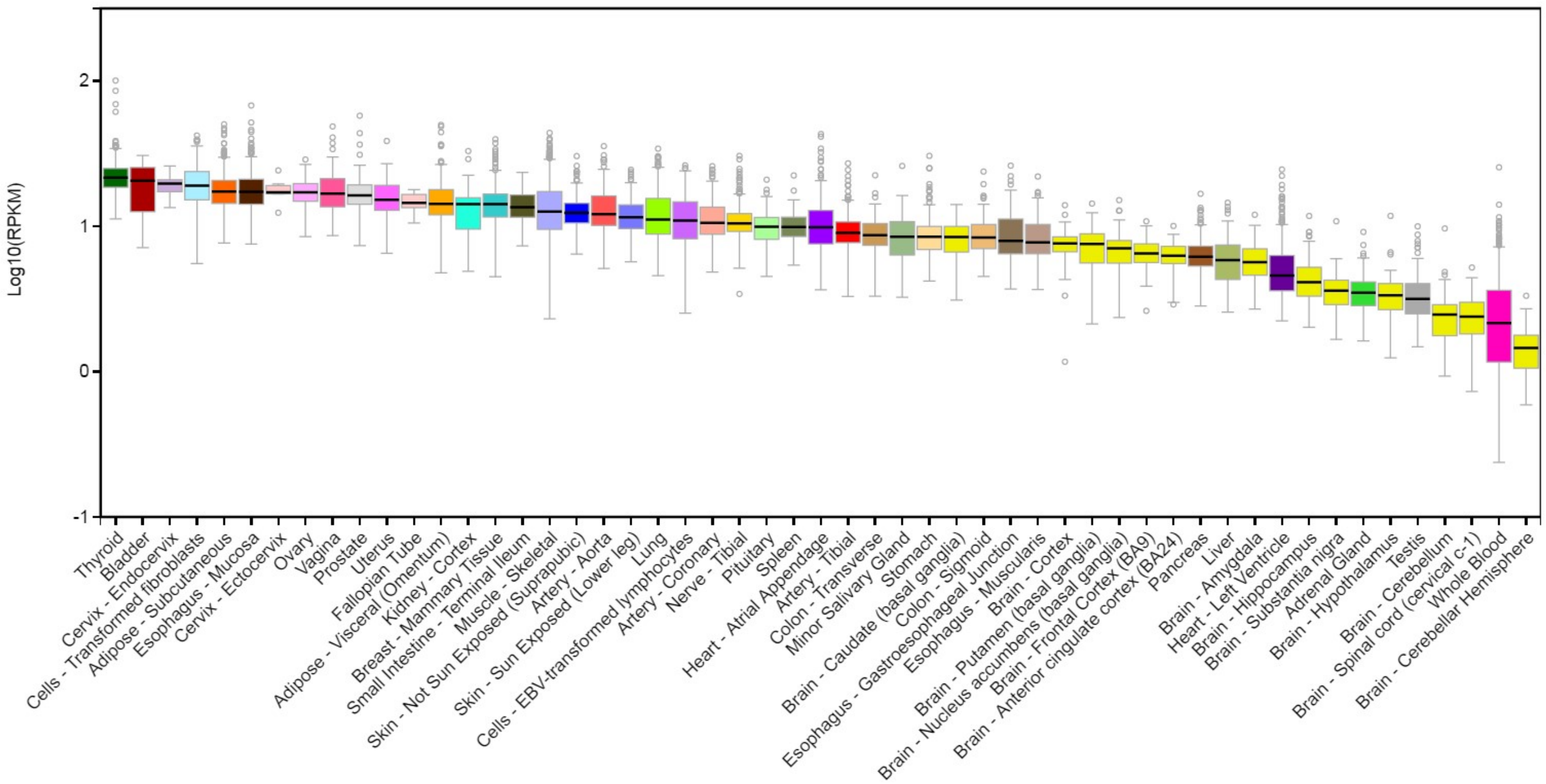

Supplementary Figure 5. Expression levels of *SMAD3* in a collection of tissues from the GTEx consortium

Expression values are shown in RPKM (Reads Per Kilobase of transcript per Million mapped reads), calculated from a gene model with isoforms collapsed to a single gene. No other normalization steps have been applied. Box plots are shown as median and 25th and 75th percentiles; points are displayed as outliers if they are above or below 1.5 times the interquartile range indicated by the whiskers. For further details, see the GTEx homepage: **(**date accessed July 6th, 2016; http://www.gtexportal.org/home/**)**.

Supplementary Table 1. Information about samples included in GWAS and meta-analysis for each study group.

Study group	*Patients*				*Controls*			
	**Count (n)**	**Average age at diagnosis (years)**	**Females (n)**	**Fraction females**	**Count (n)**	**Average age at inclusion (years)**	**Females (n)**	**Fraction females**
Iceland	1,003	53	714	71%	278,991	57	135,743	49%
Houston, USA	250	45	173	69%	363	53	202	56%
Columbus, USA	1,580	43	1,175	74%	1,628	45	1,204	74%
The Netherlands	85	40	60	71%	4,956	55	2,725	55%
Spain	83	49	65	78%	1,612	47	822	51%
**Total**	**3,001**				**287,550**			

Supplementary Table 2. Association results from the meta-analysis for five previously reported thyroid cancer risk loci

Locus	Marker [a]	Position (bp)	OA	EA	EAF	Study group	Allelic OR (95% CI)	*P*
2q35	rs11693806	217,427,435	G	C	0.285	Iceland	1.49 (1.35, 1.64)	3.6E-14
	P_{het} = 0.0069; I^2 = 71.7				0.318	Columbus, USA	1.28 (1.15, 1.43)	1.0E-05
					0.484	Houston, USA	1.92 (1.52, 2.44)	6.7E-08
					0.266	The Netherlands	1.82 (1.30, 2.56)	5.8E-04
					0.242	Spain	1.19 (0.83, 1.69)	0.35
					0.319	All combined	1.43 (1.33, 1.54)	1.5E-24
2q35	rs966423	217,445,617	T	C	0.443	Iceland	1.32 (1.19, 1.45)	3.2E-08
	P_{het} = 0.057; I^2 = 56.3				0.453	Columbus, USA	1.19 (1.09, 1.32)	4.2E-04
					0.341	Houston, USA	1.52 (1.22, 1.89)	2.7E-04
					0.404	The Netherlands	1.75 (1.28, 2.38)	3.6E-04
					0.407	Spain	1.11 (0.81, 1.52)	0.52
					0.41	All combined	1.28 (1.20, 1.37)	8.3E-15
8p12	rs2466076	32,575,278	T	G	0.467	Iceland	1.33 (1.20, 1.47)	6.1E-09
	P_{het} = 0.41; I^2 = 0				0.528	Columbus, USA	1.27 (1.15, 1.41)	2.5E-06
					0.542	Houston, USA	1.47 (1.16, 1.89)	0.0017
					0.464	The Netherlands	1.64 (1.20, 2.22)	0.0015
					0.421	Spain	1.15 (0.84, 1.59)	0.38
					0.484	All combined	1.32 (1.23, 1.41)	1.5E-17
8p12	rs2439302	32,574,851	C	G	0.458	Iceland	1.33 (1.20, 1.47)	6.2E-09
	P_{het} = 0.71; I^2 = 0				0.525	Columbus, USA	1.27 (1.14, 1.39)	5.0E-06
					0.531	Houston, USA	1.39 (1.10, 1.79)	0.0065
					0.465	The Netherlands	1.56 (1.15, 2.13)	0.0048
					0.416	Spain	1.28 (0.93, 1.75)	0.13
					0.479	All combined	1.32 (1.23, 1.41)	5.7E-17
9q22.33	rs1588635	97,775,520	C	A	0.356	Iceland	1.61 (1.47, 1.79)	1.5E-21
	P_{het} = 0.48; I^2 = 0				0.476	Columbus, USA	1.82 (1.64, 2.00)	8.0E-32
					0.463	Houston, USA	1.72 (1.35, 2.17)	8.3E-06
					0.334	The Netherlands	1.47 (1.06, 2.00)	0.021
					0.352	Spain	1.67 (1.20, 2.33)	0.0024
					0.396	All combined	1.70 (1.59, 1.82)	2.0E-58
9q22.33	rs965513	97,793,827	G	A	0.355	Iceland	1.61 (1.45, 1.75)	4.0E-21
	P_{het} = 0.52; I^2 = 0				0.475	Columbus, USA	1.79 (1.61, 1.96)	6.7E-31
					0.468	Houston, USA	1.75 (1.39, 2.22)	3.7E-06
					0.333	The Netherlands	1.47 (1.06, 2.00)	0.020
					0.355	Spain	1.64 (1.18, 2.27)	0.0031
					0.397	All combined	1.70 (1.59, 1.79)	2.2E-57

Table continued on next page

Supplementary Table 2 cont.

Locus	Marker [a]	Position (bp)	OA	EA	EAF	Study group	Allelic OR (95% CI)	*P*
14q13.3	rs368187	36,063,370	C	G	0.523	Iceland	1.49 (1.35, 1.64)	7.2E-15
	P_{het} = 0.089; I^2 = 50.5				0.626	Columbus, USA	1.35 (1.22, 1.49)	3.0E-09
					0.615	Houston, USA	1.28 (1.00, 1.64)	0.051
					0.583	The Netherlands	1.47 (1.08, 2.00)	0.014
					0.557	Spain	0.95 (0.69, 1.30)	0.74
					0.581	All combined	1.39 (1.30, 1.47)	5.1E-23
14q13.3	rs944289	36,180,040	C	T	0.561	Iceland	1.49 (1.35, 1.65)	1.0E-14
	P_{het} = 0.031; I^2 = 62.4				0.642	Columbus, USA	1.28 (1.15, 1.41)	2.1E-06
					0.624	Houston, USA	1.17 (0.91, 1.50)	0.23
					0.614	The Netherlands	1.57 (1.15, 2.14)	0.0049
					0.576	Spain	0.99 (0.72, 1.36)	0.96
					0.603	All combined	1.35 (1.27, 1.44)	2.1E-19
14q13.3	rs116909374	36,269,155	C	T	0.0492	Iceland	1.70 (1.41, 2.06)	4.2E-08
	P_{het} = 0.85; I^2 = 0				0.0445	Columbus, USA	1.67 (1.24, 2.24)	7.4E-04
					0.0500	Houston, USA	2.51 (1.19, 5.31)	0.016
					0.0352	The Netherlands	1.30 (0.50, 3.42)	0.59
					0.0272	Spain	1.87 (0.57, 6.20)	0.30
					0.0412	All combined	1.71 (1.47, 2.00)	6.2E-12

Position is according to Build 38 of the reference genome. Effect allele (EA), other allele (OA), effect allele frequency in controls (EAF), odds ratio (OR) for effect allele with upper and lower 95% confidence intervals (CI).
[a]For all markers but rs116909374, was the imputation information score ≥0.97 and the association results shown are imputed results from the meta-analysis of the Icelandic, US (Columbus, Ohio and Houston, Texas), Dutch and Spanish study groups. For rs116909374 the combined results are made up of imputed data from Iceland (with imputation information score = 1.0) and directly generated genotypes for the other four study groups.
The pairwise correlation between marker at each locus is as follows (the marker listed latter is the previously published[3,4] one):
At 2q35 the r^2 is 0.47 between rs11693806 and rs966423.
At 8p12 the r^2 is 0.94 rs2466076 and rs2439302.
At 9q22.33 the r^2 is 0.99 between rs1588635 and rs965513.
At 14q13.3 (for the more common markers) the r^2 is 0.70 between rs368187 and rs944289.
At 14q13.3 (for the less common marker) the most significant marker in the current study is the same as previously reported (rs116909374).

Supplementary Table 3. Conditional analysis of the 2q35 and 5p15.33 loci

i) Conditional analysis results for 2q35

	Unadjusted results for rs966423_C		Results for rs966423_C adjusted on rs11693806	
**Study group**	**Allelic OR (95% CI)**	**P-value**	**Allelic OR (95% CI)**	**P-value**
Iceland	1.32 (1.19, 1.45)	3.2E-08	0.96 (0.83, 1.12)	0.62
Columbus, USA	1.19 (1.09, 1.32)	4.2E-04	1.04 (0.90, 1.20)	0.56
Houston, USA	1.52 (1.22, 1.89)	2.7E-04	1.37 (0.56, 3.39)	0.49
The Netherlands	1.75 (1.28, 2.38)	3.6E-04	1.51 (0.95, 2.40)	0.082
Spain	1.11 (0.81, 1.52)	0.52	1.01 (0.66, 1.54)	0.96
All combined	1.28 (1.20, 1.37)	8.3E-15	1.03 (0.93, 1.13)	0.61

	Unadjusted results for rs11693806_C		Results for rs11693806_C adjusted on rs966423	
**Study group**	**Allelic OR (95% CI)**	**P-value**	**Allelic OR (95% CI)**	**P-value**
Iceland	1.49 (1.35, 1.64)	3.6E-14	1.41 (1.22, 1.63)	2.6E-06
Columbus, USA	1.28 (1.15, 1.43)	1.0E-05	1.24 (1.06, 1.44)	0.0066
Houston, USA	1.92 (1.52, 2.44)	6.7E-08	0.73 (0.28, 1.90)	0.52
The Netherlands	1.82 (1.30, 2.56)	5.8E-04	1.29 (0.84, 1.98)	0.24
Spain	1.19 (0.83, 1.69)	0.35	1.17 (0.74, 1.84)	0.50
All combined	1.43 (1.33, 1.54)	1.5E-24	1.31 (1.19, 1.44)	9.5E-08

ii) Conditional analysis results for 5p15.33

	Unadjusted results for rs2736100_C		Results for rs2736100_C adjusted on rs10069690	
**Study group**	**Allelic OR (95% CI)**	**P-value**	**Allelic OR (95% CI)**	**P-value**
Iceland	1.15 (1.04, 1.27)	6.5E-03	1.05 (0.94, 1.17)	0.36
Columbus, USA	1.12 (1.01, 1.23)	0.025	1.09 (0.97, 1.22)	0.15
Houston, USA	0.93 (0.74, 1.16)	0.49	1.16 (0.51, 2.67)	0.72
The Netherlands	1.25 (0.93, 1.69)	0.14	1.15 (0.81, 1.63)	0.44
Spain	1.04 (0.76, 1.43)	0.82	0.84 (0.57, 1.23)	0.36
All combined	1.11 (1.04, 1.19)	7.3E-04	1.06 (0.99, 1.15)	0.11

	Unadjusted results for rs10069690_T		Results for rs10069690_T adjusted on rs2736100	
**Study group**	**Allelic OR (95% CI)**	**P-value**	**Allelic OR (95% CI)**	**P-value**
Iceland	1.25 (1.12, 1.40)	4.8E-05	1.23 (1.09, 1.39)	6.7E-04
Columbus, USA	1.12 (1.00, 1.25)	0.043	1.07 (0.94, 1.22)	0.28
Houston, USA	1.21 (0.94, 1.57)	0.14	1.91 (0.65, 5.58)	0.24
The Netherlands	1.38 (0.97, 1.96)	0.074	1.26 (0.86, 1.85)	0.23
Spain	1.40 (0.99, 1.98)	0.059	1.53 (1.02, 2.30)	0.039
All combined	1.20 (1.12, 1.29)	3.2E-07	1.18 (1.08, 1.28)	1.3E-04

Shown are association results from the meta-analysis before and after conditional analysis, the allelic odd ratio (OR) with 95% confidence intervals and the P-value. The correlation (r^2) in Iceland between rs966423 and rs11693806 is 0.47; and between rs10069690 and rs2736100 is 0.19.

Supplementary Table 4. Association results for thyroid cancer risk variants and serum levels of thyroid related hormones.

a) Results for the six risk loci reported in the current study

				TSH		***fT3***		***fT4***	
**Locus**	**Marker (EA)**	**Chr**	**Position (bp)**	**Effect (s.d.)**	**P**	**Effect (s.d.)**	**P**	**Effect (s.d.)**	**P**
1q42.2	rs12129938 (A)	1	233,276,815	0.000	0.96	0.004	0.54	-0.003	0.59
3q26.2	rs6793295 (T)	3	169,800,667	-0.006	0.20	0.000	0.96	-0.007	0.11
5p15.33	rs10069690 (T)	5	1,279,675	0.004	0.38	0.009	0.16	0.007	0.13
5q22.1	rs73227498 (A)	5	112,150,207	-0.010	0.054	0.000	0.95	0.014	0.010
10q24.33	rs7902587 (T)	10	103,934,543	0.001	0.90	0.007	0.44	0.006	0.34
15q22.33	rs56062135 (T)	15	67,163,292	-0.006	0.18	-0.003	0.63	0.001	0.90
15q22.33	rs2289261 (C)	15	67,165,147	-0.002	0.63	0.000	0.93	0.006	0.12

b) Results for strongest variants in the current study at the five previously reported[3,4] thyroid cancer risk loci

				TSH		***fT3***		***fT4***	
**Locus**	**Marker (EA)**	**Chr**	**Position (bp)**	**Effect (s.d.)**	**P**	**Effect (s.d.)**	**P**	**Effect (s.d.)**	**P**
2q35	rs11693806 (C)	2	217,427,435	-0.021	4.6E-07	-0.005	0.35	0.019	1.8E-05
8p12	rs2466076 (G)	8	32,575,278	-0.037	2.1E-23	0.003	0.55	0.011	0.0047
9q22.33	rs1588635 (A)	9	97,775,520	-0.087	2.5E-113	0.038	2.2E-12	-0.049	2.4E-33
14q13.3	rs368187 (G)	14	36,063,370	-0.023	3.7E-10	0.006	0.26	0.012	0.0015
14q13.3	rs116909374 (T)	14	36,269,155	-0.139	1.2E-59	0.014	0.23	0.061	1.5E-11

Quantitative trait locus (QTL) association results are for the effect-allele (EA) of all markers. Shown are: the chromosome (Chr), the position in base pairs (bp) in Build 38, the P-value (P), and the effect size for serum levels of: thyroid stimulating hormone (TSH), free-triiodothyronine (fT3), and free-thyroxine (fT4). The effect size is measured in standard-deviation units (s.d.). The minus (-) sign in front of the effect size stands for a decreasing effect whereas no sign stands for an increasing effect. The QTL association results are based on measurements for the following number of Icelanders: TSH = 188,057 individuals; fT3 = 51,047 individuals; fT4 = 120,879 individuals.

References

1. International HapMap, C. *et al.* A second generation human haplotype map of over 3.1 million SNPs. *Nature* **449**, 851-61 (2007).
2. Pruim, R.J. *et al.* LocusZoom: regional visualization of genome-wide association scan results. *Bioinformatics* **26**, 2336-7 (2010).
3. Gudmundsson, J. *et al.* Discovery of common variants associated with low TSH levels and thyroid cancer risk. *Nat Genet* **44**, 319-22 (2012).
4. Gudmundsson, J. *et al.* Common variants on 9q22.33 and 14q13.3 predispose to thyroid cancer in European populations. *Nat Genet* **41**, 460-4 (2009).
